# Removal of Cr(vi) in wastewater by Fe–Mn oxide loaded sludge biochar†

**DOI:** 10.1039/d4ra00169a

**Published:** 2024-04-12

**Authors:** Chaoyang Yu, Jinyan Yang

**Affiliations:** a College of Architecture and Environment, Sichuan University Chengdu 610041 China yanyang@scu.edu.cn; b Sichuan-Tibet Railway Co., Ltd Chengdu 610041 China

## Abstract

Sludge biochar loaded with Fe–Mn oxides (FMBC) was prepared and employed to remove Cr(vi) from wastewater. The influences of solution pH, co-existing ion, contact time, adsorption temperature and Cd(vi) concentrations on removing Cr(vi) by FMBC were investigated. The Cr(vi) adsorption on FMBC had strong pH dependence. Additionally, Na^+^, Mg^2+^, Ca^2+^, SiO_3_^2−^, NO_3_^−^ and Cl^−^ ions exhibited no influence on Cr(vi) removal efficiency for FMBC, whereas there were inhibition effects of Pb^2+^, Cu^2+^, Ni^2+^, CO_3_^2−^, SO_4_^2−^, and PO_4_^3−^ on removing Cr(vi). The Cr(vi) adsorption from solution for FMBC was well described by models of pseudo-second-order and Langmuir, and the largest Cr(vi) removal capacity of FMBC reached 172.3 mg g^−1^. FMBC had good capacity for treating electroplating wastewater and mineral dissolving wastewater containing Cr(vi). After five regenerations, the 50 and 5 mg L^−1^ Cr(vi) removing efficiency of FMBC was 82.34% and 97.68%, respectively. The Cr(vi) removal for FMBC involved adsorption-reduction and re-adsorption of Cr(iii) generated by reduction. These results indicated that FMBC has good prospects for remediating Cr(vi)-containing wastewater.

## Abstract 2

Sludge biochar loaded with Fe–Mn oxides (FMBC) was prepared and employed to remove Cr(vi) from wastewater.

## Graphical abstract

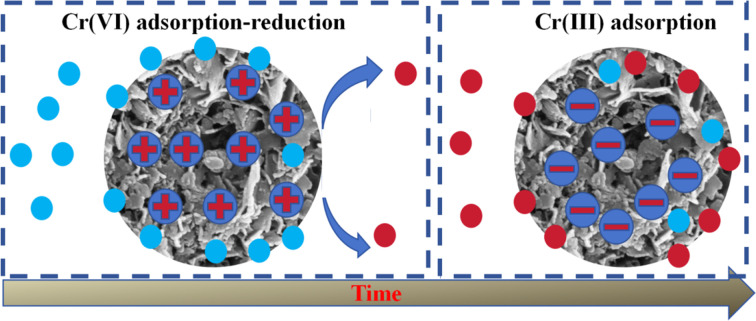

## 1 Introduction

Chromium (Cr) mainly exists in two valence states, Cr(vi) and Cr(iii), of which Cr(vi) is highly toxic, difficult to biodegrade and easy to be enriched in the food chain.^1,2^ If Cr-containing wastewater is not treated properly, it will bring great harm to human beings and nature. Currently, the main method for Cr(vi) removal is reverse osmosis, ion exchange, oxidation reduction, membrane separation and adsorption.^3^ Adsorption is considered to be a preferred method for treating Cr(vi) in wastewater because of its simplicity, low cost and high efficiency.^4,5^ Therefore, research and development of an economical, simple and efficient new adsorbent is of high application value.

Biochar prepared from waste biomass by pyrolysis has the advantages of porous structure, rich oxygen-containing functional groups, low cost and wide adaptability, which is considered as a good adsorbent material.^6–8^ However, the physicochemical properties of biochar, such as surface functional groups, porosity and specific surface area, have great influence on removing Cr(vi).^6,9^ Therefore, the Cr(vi) removal effect on biochar in water can be improved by modifying its physicochemical properties and surface structure. Previous literature showed that iron oxides increased the positive charge of biochar and its electrostatic attraction with anionic pollutants was enhanced.^10,11^ While manganese oxides hold chemically stable and rich –OH functional groups, it is an adsorbent material for efficient adsorption of contaminants in solution.^12^ However, in practical applications, the iron and manganese oxides exhibit insufficient mechanical strength, poor flocculation and easy agglomeration.^13^ Biochar as a carrier could effectively improve the agglomeration of Fe–Mn oxides. After the introduction of Fe–Mn oxides, the amount of active site of biochar increased, and its removal performance for contaminants was greatly improved.^14^ Additionally, the multi-metal materials have synergistic adsorption and reduction effects, which can improve the effect of Cr(vi) treatment.^13^ Applying composites to treat Cr(vi)-containing wastewater is expected to achieve the dual purpose of waste biomass resource utilization and Cr(vi) pollutant control.

Hence, sludge biochar loaded with Fe–Mn oxides (FMBC) was employed to remove Cr(vi) from wastewater. The intentions of this research were to: (i) investigate removal performance of FMBC for Cr(vi) in solution, (ii) elucidate the adsorption mechanism of Cr(vi) by FMBC, and (iii) validate potential of FMBC for remediating electroplating wastewater and mineral dissolving wastewater containing Cr(vi).

## 2 Materials and methods

### 2.1 Reagents

Ferric chloride (FeCl_3_·6H_2_O), manganese chloride (MnCl_2_·5H_2_O), sodium hydroxide (NaOH), hydrochloric acid (HCl), and potassium dichromate (K_2_Cr_2_O_7_) were purchased from Sinopharm Chemical Reagent Co. Ltd (Shanghai, China). Above reagent was of analytical reagent. Different concentration of Cr(vi) solutions was obtained through K_2_Cr_2_O_7_ solids dissolution. Dewatering sludge from Chengdu (Sichuan Province, China) municipal wastewater treatment plant.

### 2.2 Preparation of Fe–Mn oxide loaded sludge biochar

Air-dried dry sludge was ground into powder by ball mill. Five gram powdered dry sludges were incorporated into 100 mL deionized water and homogenously stirred for 60 min. Meanwhile, 2 g of FeCl_3_·6H_2_O and 2 g of MnCl_2_·5H_2_O were incorporated into 100 mL deionized water. Above two solutions were added to 500 mL conical flask and solution pH was adjusted to 10–11.0 by 0.1 mol L^−1^ NaOH solution. Subsequently, suspension shake in a 25 °C thermostatic oscillator at 120 rpm for 12 h. And then, the solution was filtered through a 0.23 μm filter membrane and the solids on membrane were assembled. Then, solids were dried in a thermostatic oven at 80 °C for 12 h. Thereafter, 5 g solid was placed on a ceramic boat and the boat was pyrolyzed in a muffle furnace with N_2_ atmosphere at 450 °C for 2 h. After pyrolysis, the black solid on the boat was washed with ultrapure water and anhydrous ethanol for 3 times, and the obtained black solid was dried in a constant temperature oven at 80 °C for 12 h. The dried black solid was stored in a sealed bag and recorded as FMBC.

### 2.3 Batch adsorption experiment

Adsorption experiment performed in 100 mL polyethylene centrifuge tube containing 50 mL of Cr(vi) solution. The solution pH was adjusted by 0.1 mol L^−1^ HNO_3_ or NaOH. During the reaction, the centrifuge tubes was set into a shaker at 25 °C and shaken at 150 rpm for 300 min. The effect of pH and co-existing ion on removing Cr(vi) was researched.

Adsorption isotherm: 0.4 g L^−1^ adsorbent was added to Cr(vi) solution at concentrations of 50–150 mg L^−1^, and then solution pH set to 3.0. The suspension was placed into a temperature-controlled shaker at multiple temperature (288 K, 298 K and 308 K) to reaction for 300 min. Langmuir, Freundlich, Temkin and Sips model was employed to analyze experimental result (ESI†).

Adsorption kinetics: series of centrifuge tubes containing 50 mg L^−1^ Cr(vi) solution were set up according to the adsorption time, and 0.4 g L^−1^ of adsorbent was added to the tubes. The solution pH was set to 3.0, and then adsorption experiment was carried out for 5–300 min in a thermostatic oscillator at 25 °C. The data was analyzed by pseudo-first-order kinetic model, pseudo-second-order kinetic model, Elovich model, intra-particle diffusion model and liquid film diffusion model, respectively (ESI†).

To explore practicality of FMBC, Cr(vi) removal experiments were carried out in electroplating wastewater and mineral dissolving wastewater. The content of Cr(vi) in electroplating wastewater and mineral dissolving wastewater were 40.21 and 1.35 mg L^−1^, respectively. Since pH of mineral dissolving wastewater was 6.76, the mixed solution pH of mineral dissolving wastewater and adsorbent was adjusted to 3.0 before adsorption. Adsorbent was added to 50 mL electroplating wastewater or mineral dissolving wastewater, and the mixed solution was transferred to oscillator at 25 °C and 150 rpm to adsorb 300 min. The electroplating wastewater came from Baohe swan electroplating plant (Chengdu, China) and mineral dissolving wastewater originated during the construction of railway tunnels in southwestern China. The properties of electroplating wastewater and mineral dissolving wastewater were displayed in Table S1.†

Repeatability experiments: 50 mg L^−1^ Cr(vi) solutions and 0.4 g L^−1^ absorbent were added into a centrifuge tube, and pH of suspension was set to 3.0. Then suspension was transferred to a oscillator at 25 °C and 150 rpm to adsorb for 300 min. After reaction, the solution was filtered, and the absorbent was collected on a nylon filter membrane. The sample was mixed with 0.1 mol L^−1^ NaOH, and suspension pH was probably around 10.0. The suspensions were shaken in oscillator (25 °C and 150 rpm) for 12 h. Then suspension was filtered through 0.45 μm nylon membrane filter and rinsed with ultrapure water until the rinsing solution was neutral, and samples were dried at 80 °C for 12 h. Repeatability experiment was repeated for 5 times with the regenerated absorbent and Cr(vi) adsorption performances by FMBC for each regeneration were calculated.

The total Cr contents of residual liquid were determined through an inductively coupled plasma optical emission spectrometer (ICP-OES, FL1008M018, Cary, USA). The Cr(vi) contents were determined through modified 1,5-diphenylcarbazide method.^10^ The Cr(iii) content was calculated by content difference between the total Cr and Cr(vi). The removal efficiency (eqn (1)) and adsorption capacity (eqn (2)) were counted.1
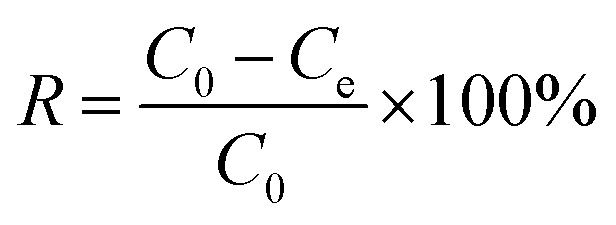
2
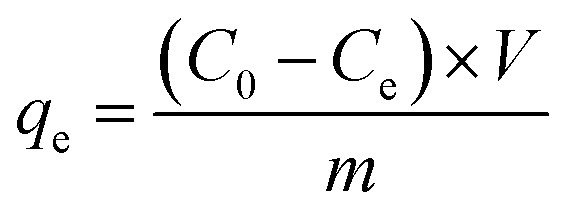
where *R* is the removal efficiency, %; *C*_0_ is the initial Cr(vi) concentration, mg L^−1^; *C*_e_ is the Cr(vi) concentration after adsorption equilibrium, mg L^−1^; *q*_e_ is the Cr(vi) adsorption capacity of the adsorbent, mg g^−1^; *V* is the solution volume, *L*; and *m* is the mass of the adsorbent, g.

## 3 Results and discussion

### 3.1 Characterization analysis

BC and FMBC surface morphology structure was illustrated in Fig. S1.† The surface of BC was smooth and flat as shown in Fig. S1a.† Nevertheless, the Fig. S1b† showed the presence of flower-like structure on the FMBC surface, which indicated that Fe–Mn compounds were successfully loaded on sludge biochar surface. According to EDS analysis, the content of Fe and Mn on the FMBC surface was higher than that of BC. Furthermore, Fig. S1c† showed that Fe and Mn were dispersed on FMBC surface, which also indicated that Fe–Mn oxide was loaded on FMBC surface.

**Fig. 1:**
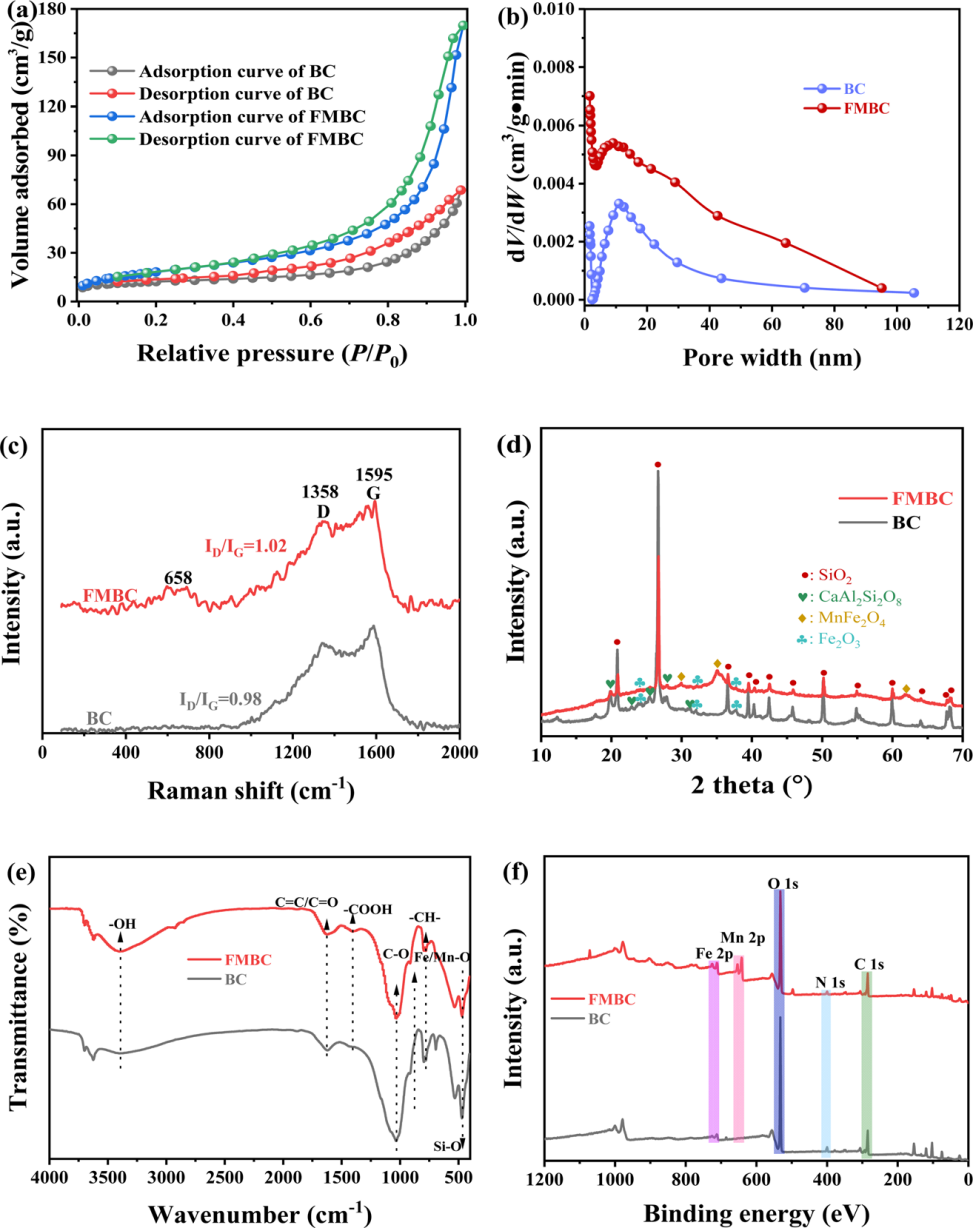
The spectra of N_2_ adsorption–desorption curves (a), pore size distribution (b), Raman (c), XRD (d), FTIR (e) and XPS (f) of BC and FMBC sample.

The N_2_ adsorption–desorption curves of BC and FMBC are displayed in Fig. 1a. Adsorption capacity of N_2_ by BC and FMBC gradually increased with increasing relative pressure *P*/*P*_0_ and a hysteresis loop was formed, which suggested that N_2_ adsorption–desorption curves for BC and FMBC was categorized as type IV isotherm.^10,15^ The non-coincidence in the second half of the adsorption–desorption curves attributed to multilayer filling influence of capillary pore, suggesting that both micropores and mesopores existed in BC and FMBC, but the material was mainly mesoporous.^16^ The specific surface area of BC and FMBC was calculated as 25.28 m^2^ g^−1^ and 67.34 m^2^ g^−1^, respectively. The pore width of BC and FMBC is displayed in Fig. 1b. The pore widths were concentrated in 2–50 nm, which suggested that BC and FMBC were mesoporous material.^3^ Moreover, the average pore size of BC and FMBC was calculated as 9.43 nm and 15.60 nm by methods, respectively.

As shown in Fig. 1c, the Raman shift at 1358 cm^−1^ and 1595 cm^−1^ was denoted as D peak and G peak, respectively.^10^ The *I*_D_/*I*_G_ ratio of BC and FMBC was calculated to be 0.98 and 1.02, respectively, and this result indicated that the graphitization of sludge biochar decreased after loading with Fe–Mn oxides. Furthermore, FMBC exhibited a new vibrational peak at 658 cm^−1^, which was considered to be the characteristic peak of MnFe_2_O_4_.^17^ The crystalline structures in BC and FMBC were investigated through XRD technique and result are displayed in Fig. 1d. In XRD plot, the main substances in BC and FMBC were SiO_2_ and CaAl_2_Si_2_O_8_, which was in agreement with previous findings.^10,18^ Moreover, the characteristic peak of Fe_2_O_3_ was detected in BC. Notably, new characteristic peaks in FMBC appeared at 2*θ* = 29.86°, 35.14° and 61.92°, which was speculated that the new characteristic peaks may be caused by MnFe_2_O_4_.^19^ In the FTIR profile of BC and FMBC (Fig. 1e), the broad band observed at 3300–3500 cm^−1^ was related to O–H stretching vibration, and characteristic peak at 1620 cm^−1^ corresponded to the vibration of C=C/C=O deformation conjugation vibration.^9^ The vibrational peak appearing at 1430 cm^−1^ was considered to be due to the –COOH group, while the vibrational peak at 1070 cm^−1^ was considered to be due to the C–O group.^20^ Notably, the intensity of O–H, C=C/C=O, and –COOH groups on FMBC were all enhanced upon loading of Fe–Mn oxides, suggesting that the Fe–Mn oxide loading increased the number of O-containing groups in the adsorbents.^13^ Additionally, the vibrational peak at 531 cm^−1^ corresponded to Fe–O group in BC.^19^ After Fe–Mn oxide loading, the characteristic peak at 531 cm^−1^ was shifted to 539 cm^−1^, which may be caused by Fe/Mn–O characteristic peak formed by Fe–Mn oxide loading.^19^

According to the full spectrum analysis by XPS (Fig. 1f), the elements C, O, N and Fe appeared in the full XPS spectrum of BC, while the element Mn was found in FMBC. In the O 1 s profiles of BC (Fig. S2a†) and FMBC (Fig. S2b†), O 1 s could be classified into M–O (M denoted Fe or Mn), C=O and C–O characteristic peaks. After loading Fe–Mn oxides, the relative contents of Fe–O/Mn–O and C=O in FMBC increased from 6.04% and 0.72% to 21.68% and 3.02%, respectively, which demonstrated that Fe–Mn oxides effectively increased the number of O-containing groups on FMBC.^13^ In Fe 2p profile of BC (Fig. S2c†), The element Fe in BC existed as Fe(iii) corresponding to binding energies of 711.07 eV and 724.57 eV.^21^ This was consistent with the results of the XRD analyses. Nevertheless, elemental Fe in FMBC (Fig. S2d†) was present in the form of both Fe(ii) and Fe(iii), and the relative contents of Fe(ii) and Fe(iii) were 44.74% and 55.26%, respectively.^21^ No characteristic peaks were detected in the fine XPS mapping of high-resolution Mn 2p in BC (Fig. S2e†). In Fig. S2f,† a distinct Mn 2p characteristic peak appeared in FMBC, and Mn 2p peaks existed in both Mn(ii) and Mn(iii) valence states.^12^ The binding energies of Mn(ii) and Mn(iii) in the Mn 2p peaks were 640.76/652.28 eV and 642.72/653.88 eV, respectively, and the relative contents of Mn(ii) and Mn(iii) were 64.28% and 35.72%, respectively.^12^ The above analyses demonstrated that the loading of Fe–Mn oxides provided developed pore structure and more O-containing groups for FMBC.

### 3.2 Influence of solution pH and co-existing ion on removing Cr(vi)

**Fig. 2:**
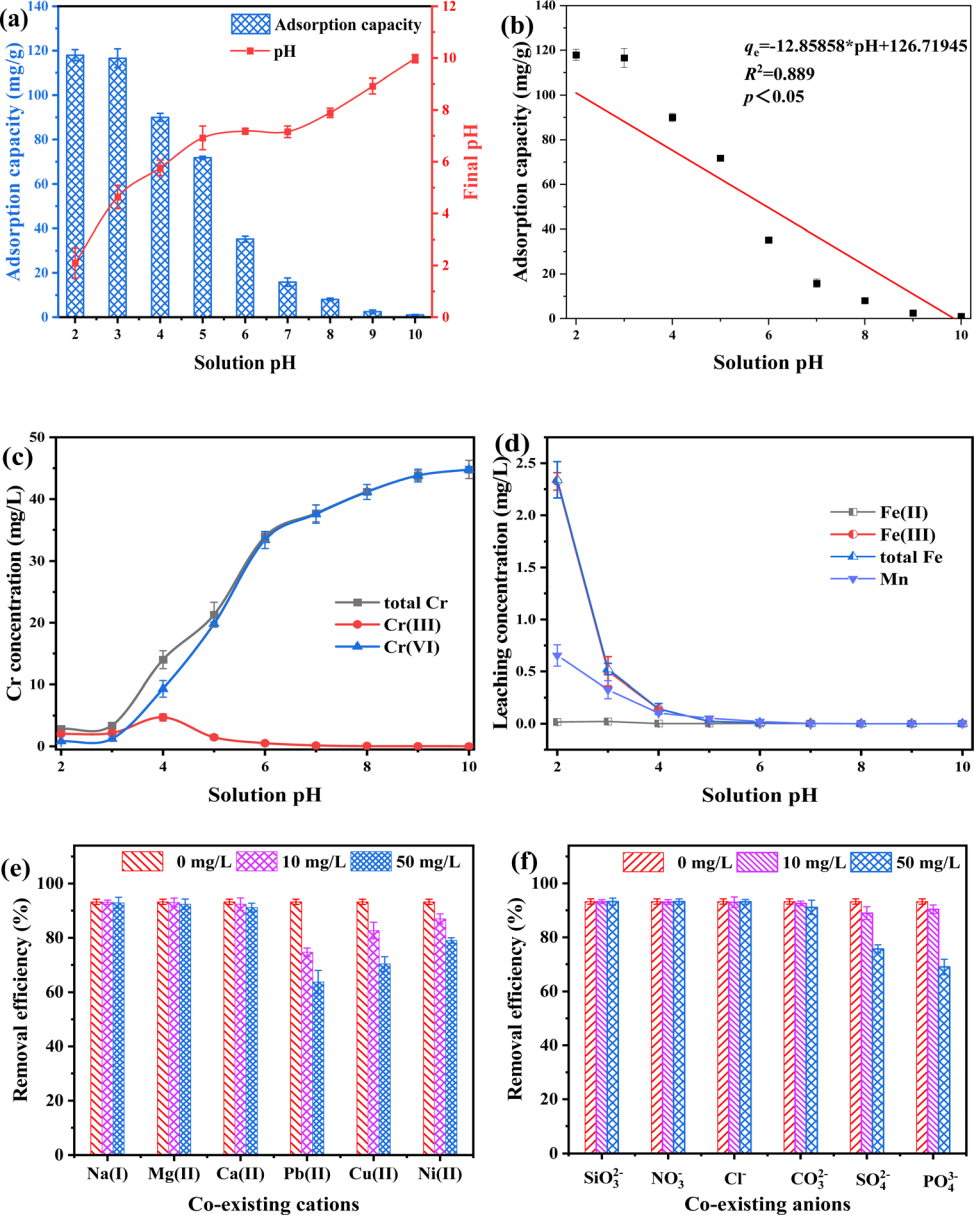
Influence of solution pH on removing Cr(vi) for FMBC and solution pH after adsorption equilibrium (a), correlation between solution pH and adsorption capacity (b), and the content of Cr, Fe and Mn in solution after adsorption equilibrium ((c) Cr and (d) Fe and Mn). Effect of co-existing cation (e) and co-existing anion (f) on Cr(vi) removal by FMBC.

Solution pH is one of crucial factor affecting contaminant removal capacity by absorbent. The result indicated that solution pH obviously (*p* < 0.05) affected Cr(vi) adsorption performance for FMBC (Fig. 2a and b). The Cr(vi) adsorption capacities for FMBC were approximately 117 mg g^−1^ at solution pH of 2.0 and 3.0, while Cr(vi) adsorption capacity by FMBC decreased to 1.03 mg g^−1^ with increasing solution pH to 10.0. The adsorption capacity of Cr(vi) by BC was considerably lower compared to FMBC (Fig. S3†). The Cr(vi) adsorption capacities for BC were about 63 mg g^−1^ at solution pH of 2.0 and 3.0, and decreased to 0.7 mg g^−1^ as the solution pH increased to 10.0. This result indicated the loading of Fe–Mn oxides improved the removal capacity of sludge biochar for Cr(vi).^13^ It was not difficult to discover that the removal performance of Cr(vi) by FMBC was stronger on acidic condition. Furthermore, final pH at adsorption equilibrium on acidic condition was greater than initial solution pH, whereas final pH was below initial pH on alkaline condition.

The contents of total Cr, Cr(iii) and Cr(vi) in residual solution were determined. As solution pH rose from 2.0 to 10.0, the concentrations of total Cr and Cr(vi) rose from 2.84 and 0.83 mg L^−1^ to 44.79 and 44.79 mg L^−1^, respectively, whereas the content of Cr(iii) increased from 2.01 mg L^−1^ to 4.70 mg L^−1^ and then decreased to under detect limit with increasing solution pH, which indicated that FMBC reduced partial Cr(vi) to Cr(iii) (Fig. 2c). More importantly, the Cr(iii) concentrations were greater than that of Cr(vi) under acidic condition, while the Cr(iii) concentration were very low small or even below the detection value under alkaline condition, which indicated that it was difficult for FMBC to reduce Cr(vi) to Cr(iii) on alkaline condition, whereas Cr(vi) ions were adsorbed through adsorption-reduction on acidic condition.^21^

The concentrations of Fe and Mn in solution after equilibrium were determined (Fig. 2d). At pH 2.0, the leaching contents of total iron ions were 2.34 mg L^−1^, and the contents of total Fe and Fe(iii) were substantially comparable, whereas the contents of Fe(ii) were small. This was partly due to the fact that Fe(ii) will be oxidized by dissolved oxygen, and partly owing to the ability of Fe(ii) to supply electron for reducing Cr(vi) and producing Fe(iii).^22,23^ Moreover, the change in Mn concentration decreased gradually with increasing solution pH. To reduce leaching concentrations of Fe and Mn ions and at the same time to ensure Cr(vi) adsorption performance for FMBC, solution pH of 3.0 was selected as the initial pH in subsequent experiments.

To further investigate the influences of coexisting ions on removing Cr(vi) for FMBC, effects of coexisting cations for Cr(vi) removal were explored (Fig. 2e and f). Na^+^ ions did not have obvious influence for removing Cr(vi) by FMBC. When concentrations of Ca^2+^ and Mg^2+^ rose from 0 mg L^−1^ to 50 mg L^−1^, Cr(vi) adsorption performance for FMBC decreased from 93.24% to 92.34% and 91.14%, respectively, which suggested that the presence of Ca^2+^ and Mg^2+^ exerted a slight inhibitory influence on removing Cr(vi) for FMBC. However, when the concentrations of Pb^2+^, Cu^2+^ and Ni^2+^ were increased to 50 mg L^−1^, the Cr(vi) adsorption performance for FMBC decreased to 63.68%, 70.32% and 78.96%, respectively. This indicated that there was a serious inhibitory influences of Pb^2+^, Cu^2+^ and Ni^2+^ on removing Cr(vi). Among these three heavy metal ions, the inhibitory effect of Pb^2+^ was more obvious. According to previous findings, oxygen-containing group had a much higher affinity for Pb^2+^ than the other metal elements. It was also possible that the competitive adsorption of Cr(vi) with Pb^2+^, Cu^2+^ and Ni^2+^ would compete for active site on FMBC, resulting in decreasing Cr(vi) adsorption performance.^10^

**Fig. 3:**
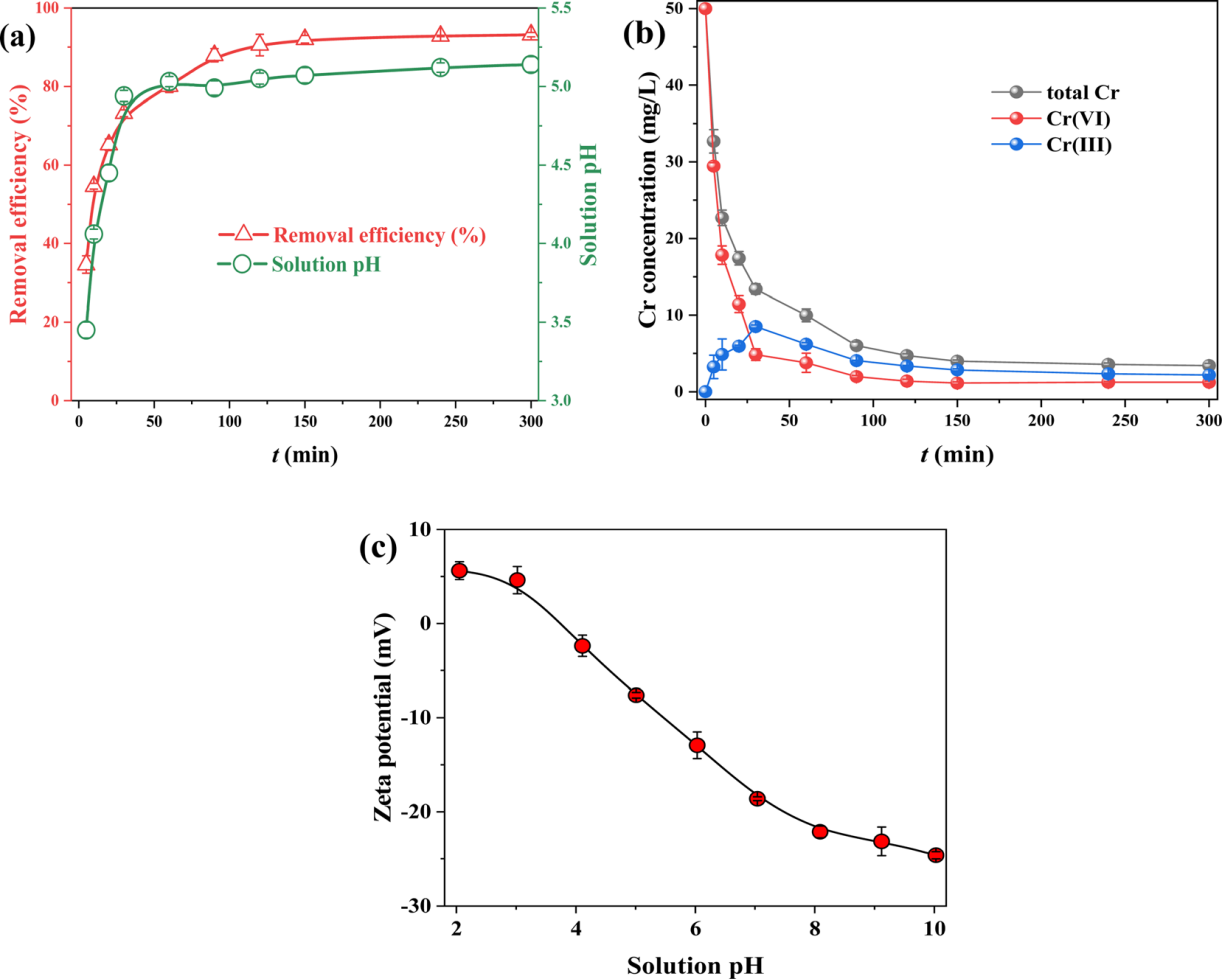
Influences of adsorption time on removing Cr(vi) for FMBC and change of solutions pH (a), influences of adsorption time on Cr concentration in solution (b) and analysis of zeta potential value of MFBC (c).

The influences of different anions on removing Cr(vi) for FMBC were examined separately (Fig. 3f). SiO_3_^2−^, NO^−^_3_ and Cl^−^ ions had no significant influences on removing Cr(vi) for FMBC when the ions concentrations rose to 50 mg L^−1^, and the removal rate was maintained at 93%. Whereas, the co-existence of CO^2-^_3_ with Cr(vi) produced a slight inhibitory influences and Cr(vi) removal performance declined to 91.08%. However, there was a significant inhibitory effect of SO_4_^2−^ and PO_4_^3−^ on removing Cr(vi) for FMBC and the adsorption performance decreased to 75.62% and 68.96%, respectively. This may be due to the fact that sulfate and phosphate ions and HCr_2_O_4_^−^ ions had similar chemical properties, which formed competitive relationship with each other to seize the active sites above FMBC.^9^

### 3.3 Adsorption kinetics

The influences of reaction time for removing Cr(vi) by FMBC are displayed in Fig. 3a. The Cr(vi) removal performance by FMBC increased rapidly from 34.62% to 73.24% within 5–30 min, and Cr(vi) removal performance by FMBC increased to 92.03% at 150 min, and then the removal efficiency stabilized at about 93% and reached equilibrium after 150 min. This was attributed to the fact that in the first 30 min, the FMBC surface contained abundant useable active site and can rapidly adsorb Cr(vi) from solution to FMBC surface.^24^ The useable active site on FMBC surface decreased dramatically during 30∼150 min, which led to slow increase in adsorption performance of Cr(vi) for FMBC. In this stage, Cr(vi) would diffuse from the FMBC surface to the inside pore channels and be adsorbed in active site of inside pore channels.^25^ The reaction reached equilibrium when all the adsorption sites inside and outside the FMBC were occupied by Cr(vi).

The Cr(vi) concentration declined rapidly from 50 mg L^−1^ to 4.86 mg L^−1^ in 0–30 min (Fig. 3b). In 30–120 min, the Cr(vi) concentration decreased slowly to 1.38 mg L^−1^ and Cr(vi) contents remained at about 1.2 mg L^−1^ after 120 min. However, the Cr(iii) concentrations increased from 0 to 8.52 mg L^−1^ then decreased to 2.18 mg L^−1^, which indicated that the removal behavior belonged to the adsorption-reduction co-action.^26^

Notably, the solution pH (Fig. 3a) first increased rapidly as the reaction proceeded, and then the solution pH remained around 5.0. Since solution pH affects presence pattern of pollutants and the electrical property of sorbent, the zeta potential value was used to represent the electrical properties of FMBC at different pH, and the result is displayed in Fig. 3c. Potentiometric analysis expressed that zeta potential value of FMBC shifted from positive to negative with the increase of pH, indicating that electrical property of FMBC shifted from positive to negative electricity.^10^ The zero potential point (pH_pzc_) of FMBC was 3.74. In aqueous solution, Cr(vi) exists mainly as the anion HCr_2_O_4_^−^ at solutions pH < 6.0, while Cr(iii) exists as Cr^3+^.^27^ This suggested that in the early stage of reaction, the positively charged FMBC adsorbed Cr(vi) to the surface by electrostatic action, and then reduced Cr(vi) to cation Cr(iii) by reducing substance, and due to electrostatic repulsion Cr(iii) ions were released to aqueous solutions.^28^ During the Cr(vi) reduction, there were a continuous consumption of H^+^ in the solution, which was the reason for the increase in pH. When solution pH outweighed pH_pzc_, FMBC adsorbed the cation Cr(iii) in solutions to surface by electrostatic action. Notably, the change in solution pH was mainly concentrated within 30 min, indicating that reduction reaction of Cr(vi) for FMBC was mainly concentrated in early stage of removal process.

**Fig. 4:**
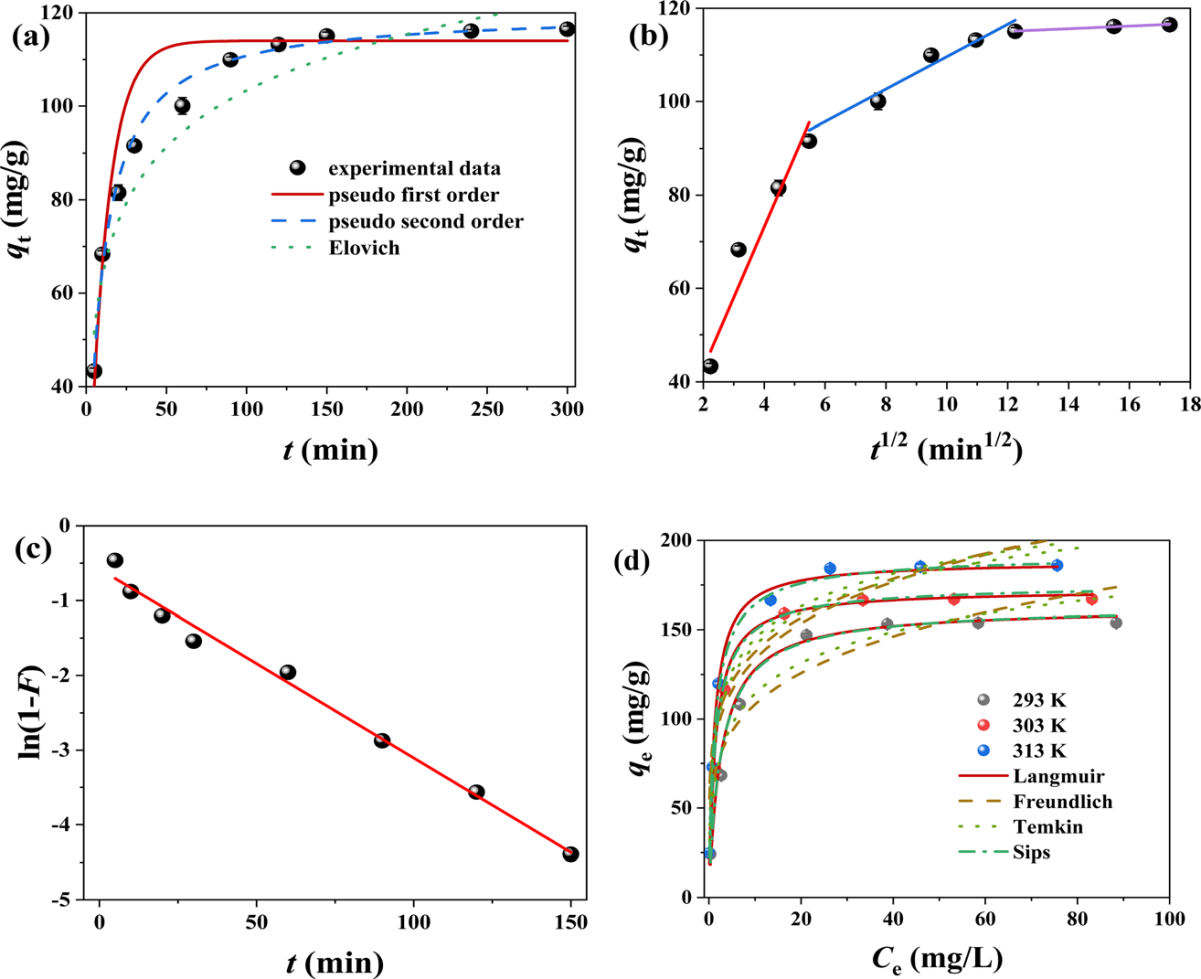
The kinetic models for removing Cr(vi) by FMBC ((a) pseudo-first-order, pseudo-second-order and Elovich, (b) intra-particle diffusion model and (c) liquid film diffusion model) and the adsorption isotherm for removing Cr(vi) by FMBC at different temperature (d).

To research removal process of Cr(vi) for FMBC, the pseudo first order kinetic model, pseudo second order kinetic model and Elovich model were chosen to describe the experiment results, and fitting result is displayed in Fig. 4a and Table 1. The linear correlation coefficient of pseudo second order kinetic model (*R*^2^ = 0.997) was higher than that of pseudo first order kinetic model (*R*^2^ = 0.967) and Elovich model (*R*^2^ = 0.941). Additionally, the difference between the theoretical adsorption capacity (*q*_e,cal_) obtained from pseudo first order kinetic model (114.00 mg g^−1^) and the actual adsorption capacity (*q*_e,exp_, 116.48 mg g^−1^) was large, whereas the difference between the fitted value from pseudo second order kinetic model (117.22 mg g^−1^) and the *q*_e,exp_ was small, which indicated that the pseudo second order kinetic model could better describe the Cr(vi) removal for FMBC than the pseudo first order kinetic model.^29^ This expressed that the removal behavior of Cr(vi) for FMBC was mainly controlled by chemisorption.^30^ Briefly, the Cr(vi) removal behavior by FMBC was primarily controlled through chemical effects (*e.g.*, complexation and electron transfer), while physical effects (*e.g.*, electrostatic effects and pore filling) were secondary to controlling the reaction process.^5,12^

**Table 1:** Adsorption kinetic model fitting parameters

Model	Parameter	Fitting result
Experimental data	*q*_e,exp_ (mg g^−1^)	116.48
Pseudo-first-order	*q*_e,cal_ (mg g^−1^)	114.00
	*k*_1_ (min^−1^)	0.086
	*R* ^2^	0.967
Pseudo-second-order	*q*_e,cal_ (mg g^−1^)	117.22
	*k*_2_ (g mg^−1^ min^−1^)	0.001
	*R* ^2^	0.997
Elovich	*α* (mg g^−1^ min^−1^)	66.723
	*β* (g mg^−1^)	0.057
	*R* ^2^	0.941

Ordinarily, diffusion behavior in removal process is determined by multiple mechanism. The intra-particle diffusion model and the liquid film diffusion model were employed to find out the diffusion behavior and the rate control steps. The curves of *q*_t_ over *t*^1/2^ consisted of three linear segments (Fig. 4b), indicating that Cr(vi) removal behavior by FMBC had multiple stage.^9^ In general, three stages were divided into external diffusion, internal diffusion and adsorption equilibrium. The curve of external diffusion stage had high slope (*K*_d1_), indicating Cr(vi) ion in solutions transferred to outer surface. The second part of diffusion trend slowed down significantly and belonged to the internal diffusion phase, where Cr ions diffused from the external surface into the internal structure of FMBC and were subsequently adsorbed to internal adsorption site.^23^ The third part was equilibrium stages. Additionally, the *C*_i_ values (Table S2†) for all stages showed a tendency to deviate from 0, suggesting that intra-particle diffusion was not the only controlling factor in removal processes.^31^ Moreover, the intra-particle diffusion constants showed *K*_d1_ > *K*_d2_ > *K*_d3_, and the boundary layer constants showed *C*_1_ < *C*_2_ < *C*_2_, which showed that external diffusion dominated the removal behavior.^10^ The outcome of film diffusion is displayed in Fig. 4c and Table S2.† The outcome indicated that liquid film diffusion diagram held linear relationship, and *R*^2^ value (0.990) kept at a high level, which indicated that the liquid film diffusion played a decisive role in the kinetic process.^9^ Briefly, the removal behavior can be divided into the following step: liquid film diffusion first controlled adsorption behavior by removing Cr(vi) from liquid phase to outer surface of FMBC; then the entire removal processed were determined by intra-particle diffusion, and Cr(vi) entered into the inner pore structure and loaded into internal active sites until adsorption saturation.

### 3.4 Adsorption isotherm

The Cr(vi) adsorption performance for FMBC rose rapidly as Cr(vi) concentration rose from 50 mg L^−1^ to 100 mg L^−1^, which attributed to large Cr(vi) concentrations difference between solutions and FMBC, which created large mass-transfer resistance and allowed Cr(vi) to be adsorbed onto FMBC surface. However, the Cr(vi) adsorption performance by FMBC did not change much as concentration rose from 100 mg L^−1^ to 150 mg L^−1^, because active sites on FMBC surface were limited.^29^

To further understand the properties of the adsorption, four commonly isotherm models (Langmuir, Freundlich, Temkin and Sips models) were used in this work, and the result is shown in Table 2 and Fig. 4d. The *R*^2^ (0.992–0.996) value of Langmuir model was significantly greater than Freundlich model (0.752–0.867), Temkin model (0.934–0.966) and Sips model (0.924–0.961), which indicated that Cr(vi) adsorption processes for FMBC were homogeneous monolayer adsorption.^32^ Additionally, the results of chi-square test (*χ*^2^) showed that Langmuir model could better describe Cr(vi) removal process for FMBC than the Freundlich, Temkin and Sips model.^12^ The separation factor (*R*_L_) of Langmuir model remained between 0 and 1 (Fig. S4a†), suggesting that Cr(vi) removal by FMBC was effective.^10^ Meanwhile, elevated temperature reduced the *R*_L_ value, which indicated that high temperature could promote Cr(vi) removal for FMBC.^10^ According to Langmuir model, the largest theoretical removal performance of FMBC at 303 K was 172.34 mg g^−1^. Temkin model (*R*^2^ > 0.93) well described the experimental results, suggesting that there was strong intermolecular force between Cr in solution and the FMBC surface, which may be involved in the redox action and complexation.

**Table 2:** Adsorption isotherm fitting parameter of removing Cr(vi) for FMBC

Model	Parameter	Temperature		
		288 K	298 K	308 K
Langmuir	*q*_max_ (mg g^−1^)	162.29	172.34	188.09
	*K*_L_ (L mg^−1^)	0.369	0.700	0.832
	*R* ^2^	0.992	0.996	0.996
	*χ* ^2^	0.132	0.485	0.632
Freundlich	*K*_f_ (mg^1−*n*^·L^*n*^ g^−1^)	65.47	89.18	83.50
	*n*	4.593	5.317	4.919
	*R* ^2^	0.867	0.752	0.829
	*χ* ^2^	22.485	12.364	36.95
Temkin	*b*_T_ (kJ mol^−1^)	92.77	95.54	97.47
	*A*_T_ (g^−1^)	9.599	21.853	24.827
	*R* ^2^	0.949	0.934	0.966
	*χ* ^2^	5.96	24.591	12.057
Sips	*Q*_m_ (L mg^−1^)	62.27	124.92	152.19
	*K*_s_	0.959	0.911	0.862
	*m*	0.381	0.711	0.790
	*R* ^2^	0.924	0.956	0.961
	*χ* ^2^	8.317	6.214	5.938

Based on the Van't Hoff equation, the ln *K*^0^ was used to plot 1/*T* (Fig. S4b†), and the slope and intercept of obtained straight line were used to calculate the value of Δ*H*^0^ and Δ*S*^0^, and then the value of Δ*G*^0^ was calculated (Table 3). The Δ*H*^0^ was greater than 0, which indicated that the removal reaction was a heat-absorbing reaction.^9^ The negative value of Δ*G*^0^ decreased with increasing temperatures, demonstrating that Cr(vi) removal behavior for FMBC was spontaneous.^16^ Δ*S*^0^ was positive, suggesting that Cr(vi) removal processes for FMBC were entropy-driven, and degree of freedom at solid–liquid interface increased during removal process.^20^

**Table 3:** Thermodynamic fitting parameter of removing Cr(vi) for FMBC

*T*/K	Δ*G*^0^ (kJ mol^−1^)	Δ*H*^0^ (J k mol^−1^)	Δ*S*^0^ (kJ mol^−1^)
288.15	−7.864		
298.15	−9.422	32.301	1.396
308.15	−10.650		

### 3.5 Reduction-adsorption mechanism

**Fig. 5:**
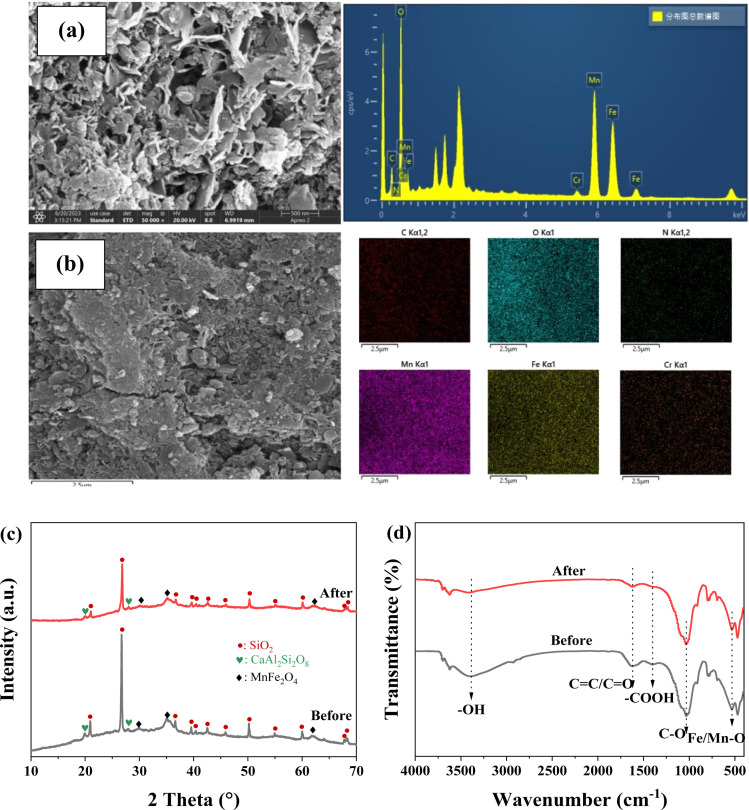
The result of SEM-EDS (a and b), FTIR (c) and XRD (d) analysis for Cr(vi) removal by FMBC.

The morphological features and surface element of FMBC were analyzed by SEM-EDS (Fig. 5a and b). After adsorption, the uneven surface became rougher than before adsorption and massive small particles were attached, which may be due to that Cr adsorbed on FMBC surface. According to the mapping pattern of EDS, elemental Cr appeared on the FMBC surface after adsorption, which demonstrated that Cr in the aqueous solution was successfully removed by FMBC. The crystalline structure (Fig. 5c) of FMBC before and after removal remained basically unchanged, and the main components were SiO_2_ and CaAl_2_Si_2_O_8_.^33,34^ However, the characteristic peaks of MnFe_2_O_4_ at 29.86°, 35.14° and 61.92° showed different degrees of decrease after adsorption, which could be the involvement of MnFe_2_O_4_ in Cr(vi) removal from aqueous solution.

To further illustrate mechanisms of Cr(vi) adsorption for FMBC, the functional groups of FMBC were analyzed by FTIR (Fig. 5d). After removal, the wavenumber of hydroxyl group –OH at 3390 cm^−1^ in FMBC shift to 3420 cm^−1^, and the intensity of –OH vibrational peaks was weakened. Notably, the intensity of vibrational peaks of –COOH group, C–O group and Mn/Fe–O group in FMBC were all weakened or shifted after adsorption. Most notably, the wavenumber of the –COOH group shifted from 1400 cm^−1^ to 1380 cm^−1^ after the adsorption, and the intensity of the vibrational peak of the –COOH group also underwent a significant weakening. The wavenumber of C–O group was offset from 1040 cm^−1^ to 1030 cm^−1^. In addition, the vibrational peak of Mn/Fe–O group could be observed to be offset from 539 cm^−1^ to 526 cm^−1^ and the intensity of the peak appeared to be slightly reduced, which could be due to the reduction caused by the dissolution of the Mn or Fe elements in FMBC. These results indicated that the oxygen-containing groups (*e.g.*, –OH, –COOH, C–O, and Mn/Fe–O groups) on the FMBC complex with negatively charged Cr(vi).^13,25,35^ Notably, the vibrational peak of C=C groups at 1640 cm^−1^ was shifted to 1610 cm^−1^, where the vibrational peaks were attributed to the C=C group on the carbon ring, which could be able to provide π-electrons as the adsorption site for contaminants and remove contaminants from solution by cation–π interactions.^32^ However, since Cr(vi) existed mainly as anion in solution, this could be the result of cationic–π interaction between Cr(iii) generated by reduction and C=C structure on FMBC during the adsorption process.^20^ According to the previous findings, the C=C group shifted from 1640 cm^−1^ to 1610 cm^−1^ after Cr(vi) removal from solution, which was also consistent with the present study.^1,22^ These analysis showed that FMBC adsorbed Cr(vi) on surface through complexation and electrostatic interactions, and Cr(vi) ion was reduced to cationic Cr(iii), and then released cation Cr(iii) into solutions by electrostatic repulsion. When solution pH outweighed zero potential point of FMBC, the negatively charged FMBC adsorbed the positively charged Cr(iii) from the solution in the form of Cr(iii)–π interaction, complexation and electrostatic interaction.

**Fig. 6:**
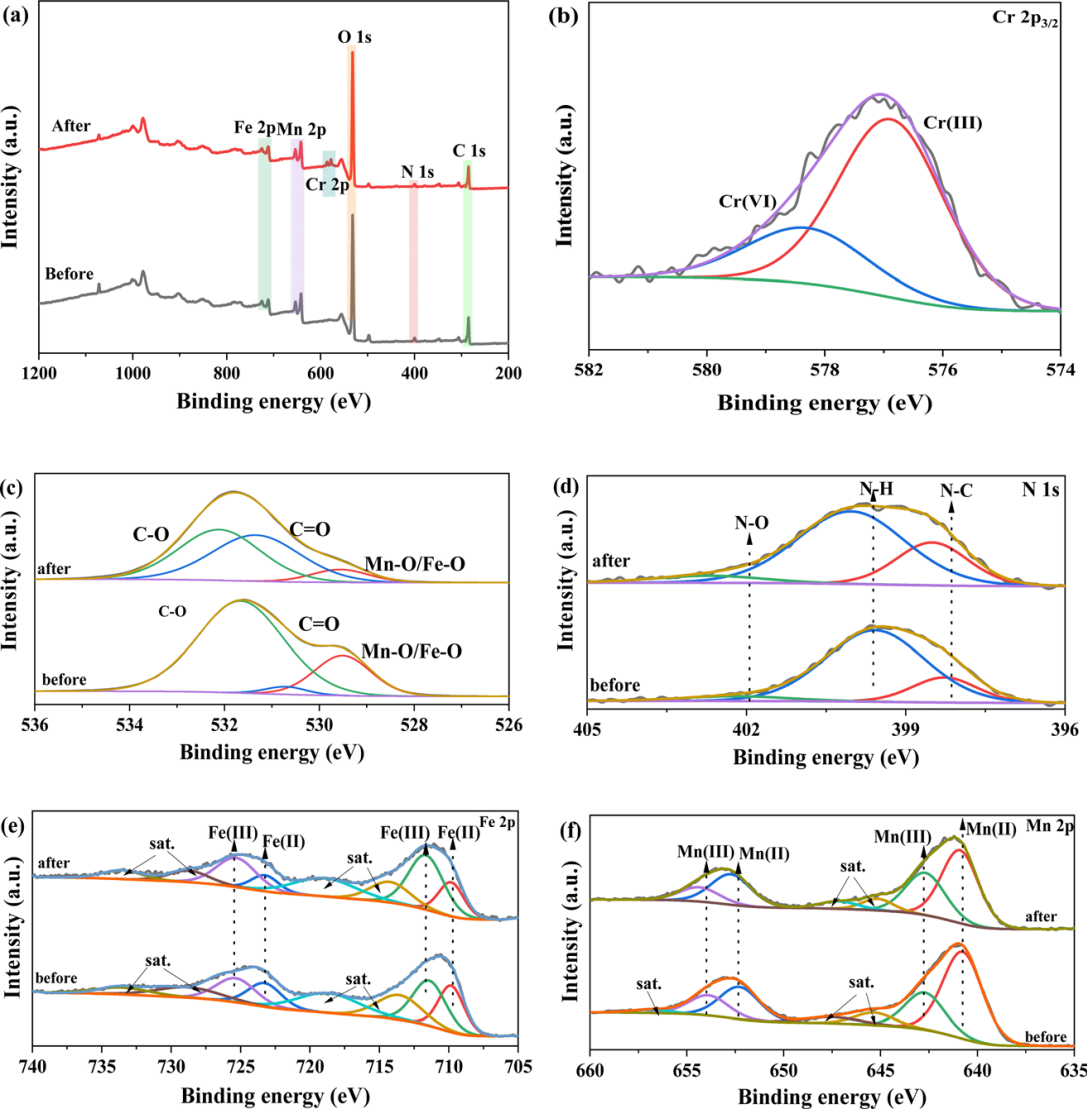
XPS analysis of FMBC after Cr(vi) removal ((a) full spectrum, (b) Cr 2p_3/2_, (c) O 1s, (d) N 1s, (e) Fe 2p and (f) Mn 2p).

To gain deeper understanding of adsorption mechanism of Cr(vi) for FMBC, the sample before and after removal was analyzed through XPS. Fig. 6a showed full spectrum of FMBC before and after Cr(vi) adsorption, and Cr 2p peaks were detected on the full spectrum after the removing Cr(vi), suggesting that Cr(vi) ions were successfully adsorbed in FMBC surface. The peak of Cr(iii) and Cr(vi) appeared in Cr 2p_3/2_ fine spectra (Fig. 6b), further indicating that Cr(vi) ions were reduced to Cr(iii) by FMBC. The Cr(iii) and Cr(vi) peaks correspond to binding energies of 576.88 eV and 578.30 eV, respectively, the percentage of Cr(iii) and Cr(vi) was 72.77% and 26.23%, respectively. This result indicated that the vast majority of Cr(vi) was removed through adsorption-reduction.^24,28^ The primary peak of O 1 s before removal in Fig. 6c were Mn/Fe–O (529.51 eV), C–O (530.71 eV) and C=O (531.66 eV).^10,19,36^ The decreasing peak areas of Mn/Fe–O/C–O and increasing peak area of C=O indicated that FMBC could be used as electrons transfer medium and attend reactions through the gain and loss of electron by O-containing functional group.^3^ In addition, binding energy of C–O and C=O appeared to increase after the reaction, which was presumed to be due to the O-containing groups in FMBC complexing with Cr(vi).^23^ In N 1 s spectra (Fig. 6d), the peaks were N–C (398.28 eV), N–H (399.58 eV) and N–O (402.20 eV).^3^ After Cr(vi) removal, the binding energy of N–C, N–H and N–O groups shifted to 398.50 eV, 400.03 eV and 402.70 eV, respectively, suggesting that chemical reaction between the N-containing groups in FMBC and Cr(vi) took place.^3^ Previous literature demonstrated the ability of N–C groups in biochar to provide lone pair electrons for coordination reactions with Cr(vi).^37^ Moreover, previous literature had also shown that the N–H group in biochar could act as an electron donor triggering an electron transfer reaction to facilitate the reduction of Cr(vi).^38^ The Fe 2p spectra (Fig. 6e) were roughly classified into Fe(ii) and Fe(iii), and the relative amount of Fe(ii) was reduced from 44.74% to 29.78% after the reaction, while the content of Fe(iii) in FMBC increased from 55.26%. This result indicated that Fe(ii) in FMBC could reduce Cr(vi) to Cr(iii).^32^ Similarly, in Mn 2p spectrum (Fig. 6f), the content of Mn(ii) decreased from 64.28% to 60.50% after the reaction, which also indicated the redox reaction between Mn(ii) and Cr(vi).^19^ The result demonstrated that Mn and Fe in FMBC could reduce Cr(vi) to Cr(iii). Meanwhile, the binding energies of both Mn and Fe shifted to higher energy levels after reaction, which further confirmed that Mn and Fe played a key role in removing Cr(vi).^13^

Thereby, removing Cr(vi) for FMBC consisted of complexation containing O/N groups, cation–π interactions, electrostatic interactions, and reduction. The reduction process was shown in the following equation:

3 3Mn^2+^ + HCrO_4_^−^ + 7H^+^ → 3Mn^3+^ + Cr^3+^ + 4H_2_O

4 3Fe^2+^ + HCrO_4_^−^ + 7H^+^ → 3Fe^3+^ + Cr^3+^ + 4H_2_O

5 6Mn^2+^ + Cr_2_O_7_^2−^ + 14H^+^ → 6Mn^3+^ + 2Cr^3+^ + 7H_2_O

6 6Fe^2+^ + Cr_2_O_7_^2−^ + 14H^+^ → 6Fe^3+^ + 2Cr^3+^ + 7H_2_O

Anionic Cr(vi) in solutions was adsorbed to FMBC surface by complexation containing N/O groups or electrostatic interaction, and Cr(vi) ions on FMBC surface were reduced to positively charged Cr(iii) by reducing substances in FMBC (*e.g.*, divalent iron/manganese or reduced phenolic hydroxyl groups). The resulting cation Cr(iii) with the positively charged FMBC would undergo electrostatic repulsion and be released into the aqueous solution. Notably, the reduction reaction continued to consume massive H^+^ in the aqueous solutions. When solutions pH was higher than pH_pzc_, Cr(iii) in the aqueous solution could be adsorbed onto the FMBC surface through the complexation of N/O groups, electrostatic interactions, and cation–π interactions.

### 3.6 Performance in treating Cr(vi)-containing wastewater

**Fig. 7:**
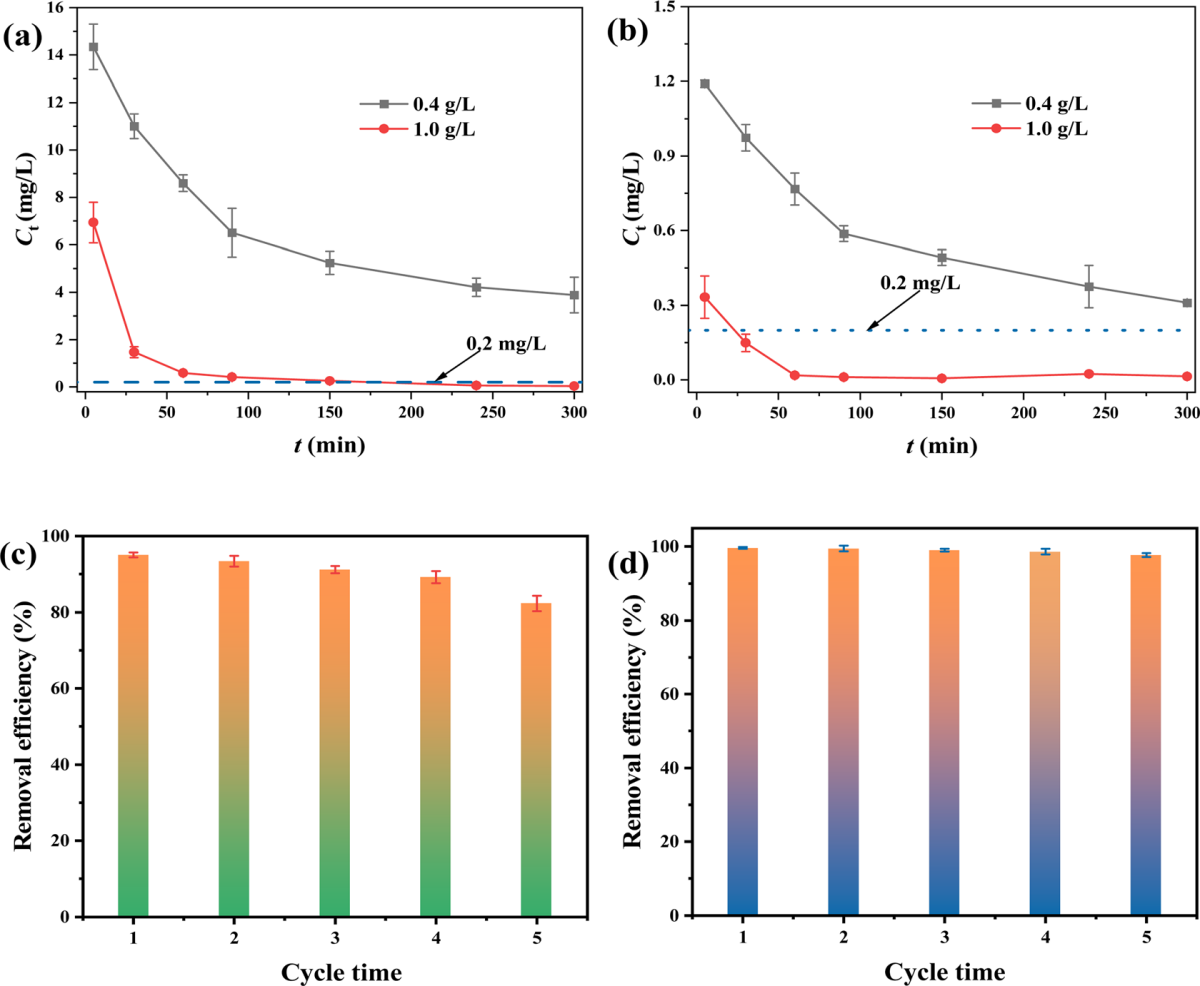
Removing Cr(vi) in electroplating wastewater (a) and dissolved mineral wastewater (b) for FMBC. Regeneration experiment of Cr(vi) removal by FMBC ((c) 50 mg L^−1^ and (d) 5 mg L^−1^).

To explore practicality, the FMBC was employed to remove Cr(vi) from electroplating wastewater (Fig. 7a) and mineral dissolving wastewater (Fig. 7b). At dosage of 0.4 g L^−1^, Cr(vi) concentration in both water samples decreased rapidly and the residual Cr(vi) contents were higher than 0.2 mg L^−1^, and this result did not meet the discharge standard for Cr(vi)-containing wastewater (0.2 mg L^−1^). To increase the removal rate, the dosage of FMBC was increased to 1 g L^−1^, and FMBC was able to remove Cr(vi) to less than 0.2 mg L^−1^ (Chinese Cr(vi) emission standard) in both electroplating wastewater and mineral dissolving wastewater at 90 min and 30 min, respectively, which indicated that FMBC had potential to be applied to the treatment of real wastewater.

### 3.7 Regeneration experiment

After each adsorption, the FMBC in aqueous solution was separated by magnet, then the adsorbed FMBC was regenerated by shock desorption for 12 h. In Fig. 7c, removal efficiency of Cr(vi) by FMBC declined with increasing number of regenerations when Cr(vi) contents were 50 mg L^−1^. However, Cr(vi) removal performance by FMBC was still 82.34% in the fifth regeneration. When Cr(vi) contents were declined to 5 mg L^−1^ (Fig. 7d), the Cr(vi) removal efficiency for FMBC was still as high as 97.68% after five regenerations. This result indicated that FMBC exhibited excellent potential to treat Cr(vi)-containing wastewater.

## 4 Conclusion

In this research, Fe–Mn oxide loaded sludge biochar (FMBC) was prepared. The Cr(vi) removal processes for FMBC were in accordance with pseudo-second-order kinetic and Langmuir model, and largest removal performance of FMBC for Cr(vi) was 172.34 mg g^−1^. Additionally, the Cr(vi) adsorption processes for FMBC were a spontaneous, monolayer and homogeneous chemical adsorption, and held obvious pH-dependent. Removing Cr(vi) mechanism for FMBC involved Cr(vi) adsorption-reduction, and the re-absorption process of Cr(iii) generated by reduction. FMBC showed excellent performance in removing Cr(vi) from electroplating wastewater and mineral dissolving wastewater. Additionally, FMBC was regenerated at least five times and effectively remove Cr(vi) in wastewater.

## Data availability

The authors confirm that the data supporting the findings of this study are available within the article and its ESI.†

## Conflicts of interest

The authors declare that we have no competing financial interests.

## Supplementary Material

RA-014-D4RA00169A-s001
