# Supplementary material for: Removal of Cr(vi) in wastewater by Fe–Mn oxide loaded sludge biochar
Source: RSC Adv. 2024 Apr 12;14(17):11746–57. doi: 10.1039/d4ra00169a (PMC11009720; doi:10.1039/d4ra00169a)
Supplement: RA-014-D4RA00169A-s001 [file RA-014-D4RA00169A-s001.pdf]

Electronic Supplementary Material (ESI) for RSC Advances.
This journal is © The Royal Society of Chemistry 2024

# Removal of Cr(VI) from wastewater by Fe-Mn oxides loaded sludge biochar

*Chaoyang Yu* [a,b], *Jinyan Yang* [a, *]

[a] *College of Architecture and Environment, Sichuan University, Chengdu 610041, China*

[b] *Sichuan-Tibet Railway Co., Ltd, Chengdu 610041, China*

* *Corresponding author: yanyang@scu.edu.cn*

## Adsorption models

### Adsorption kinetic models

The pseudo-first-order model (Eq. 1), the pseudo-second-order model (Eq. 2), Elovich model (Eq. 3), the intraparticle diffusion model (Eq. 4) and Film diffusion model (Eq. 5) were used for fitting analysis.

$$\ln(q_e - q_t) = \ln q_e - k_1 t \qquad (1)$$

$$\frac{t}{q_t} = \frac{1}{1 + q_e^2 k_2} + \frac{t}{q_e} \qquad (2)$$

$$q_t = \beta \ln(\alpha\beta) + \beta \ln(t) \qquad (3)$$

$$q_t = K_{id} t^{1/2} + C_i \qquad (4)$$

$$\ln(1 - F) = K_{fd} t + C_{fd} \qquad F = \frac{q_t}{q_e} \qquad (5)$$

where $q_e$ and $q_t$ are the adsorption capacity at the equilibrium time and time "$t$" time (mg/g), respectively; $k_1$ and $k_2$ represent adsorption rate constant of the pseudo-first-order ($min^{-1}$) and the pseudo-second-order (g/mg·min), respectively; $\alpha$ and $\beta$ are the initial absorbance (mg/g·min) and desorption constant (g/mg), respectively; $K_{id}$ [mg/(g·$min^{1/2}$)] is rate constants of intra-particle diffusion; $C_i$ is constant of the intra-particle diffusion model. $K_{fd}$ and $C_{fd}$ are rate constant and constant of film diffusion model, respectively.

### Adsorption isotherm models

The Langmuir model (Eq. 6, and Eq. 7), Freundlich model (Eq. 8), Temkin model (Eq. 9) and Sips model (Eq. 10) were used to fit these data.

$$q_e = \frac{q_{max} K_L C_e}{1 + K_L C_e}, \qquad (6)$$

$$R_L = \frac{1}{1 + K_L C_0} \qquad (7)$$

$$q_e = K_f C_e^{1/n}$$

(8)

$$q_e = \frac{RT}{b_T}\ln(A_T C_e)$$

（9）

$$q_e = \frac{Q_m(K_s C_e)^{1/m}}{1+(K_s C_e)^{1/m}} \quad (10)$$

where $q_e$ is the adsorption capacity at equilibrium (mg/g); $C_e$ is the concentration of Cr(VI) ions at adsorption equilibrium (mg/L); $q_{max}$, $K_L$ and $R_L$ are the maximum adsorption capacity of Cr(VI) ions (mg/g), Langmuir equilibrium constant (L/mg) and separation factor, respectively; $K_f$ and $n$ represent the Freundlich affinity coefficient ($mg^{1-n}\cdot L^n/g$) and Freundlich constant related to the surface site heterogeneity, respectively; $A_T$ (1/g) and $b_T$ (kJ/mol) are Temkin constants; $R$ is the global constant of gases, and $T$ is the absolute temperature (K); $K_s$ (L/mg), $C_s$ and $m$ are Sips constants.

Additionally, to ensure the correctness of the fit of the non-linear kinetic model, the chi-square test will be used to check how well the model fits. Obtained by judging the sum squares differences between the experimental and the calculated data, with each squared difference is divided by its corresponding value (calculated from the models). Small $\chi^2$ value indicates its similarities while a larger number represents the variation of the experimental data.

The thermodynamic investigation of Cd(II) removal at different temperatures (288 K, 298 K, and 308 K). The thermodynamic parameters for the removal process were calculated using Eq. (11) and Eq. (12), and the relevant parameters were listed in Table 3.

$$\ln K^0 = \frac{\Delta S^0}{R} - \frac{\Delta H^0}{RT} \quad (11)$$

$$\Delta G^0 = -RT\ln K^0 \quad (12)$$

where, $R$ is the gas constant (8.314 kJ/mol). $K^0$ is the equilibrium constant and $T$ is the Kelvin temperature (K). The $\Delta G^0$, $\Delta H^0$ and $\Delta S^0$ represent the standard free energy (kJ/mol), enthalpy (J/mol), and entropy (J/mol), respectively. The values of $\Delta H^0$ and $\Delta S^0$ were calculated from the slope and intercept of the linear regression curve of $\ln K^0$ versus $1/T$ (Fig. S2d).

Characterization

Pore structure parameters were measured by a specific surface area analyzer (BET, Tristar II Plus 2.02, USA) and analyzed by BET method. Scanning electron microscopy with energy dispersive spectrometer (SEM-EDS, JSM-7500F, JEOL, Japan). The material composition of the samples was surveyed by a X diffraction analyzer (XRD, D8X, Bruker, Germany). A X-ray photoelectron spectrometer (XPS, Escalab 250Xi, Thermo Fisher Scientific, USA) was used to analyze the elemental composition and valence changes of the samples. The functional groups were investigated using a Fourier Transform Infrared Spectroscopy (FTIR, Nicolet-460, Thermo Fisher, USA). The zeta potential of different pH solution was measured by using a zeta potential analyzer (Zetasizer, Malvern, UK).

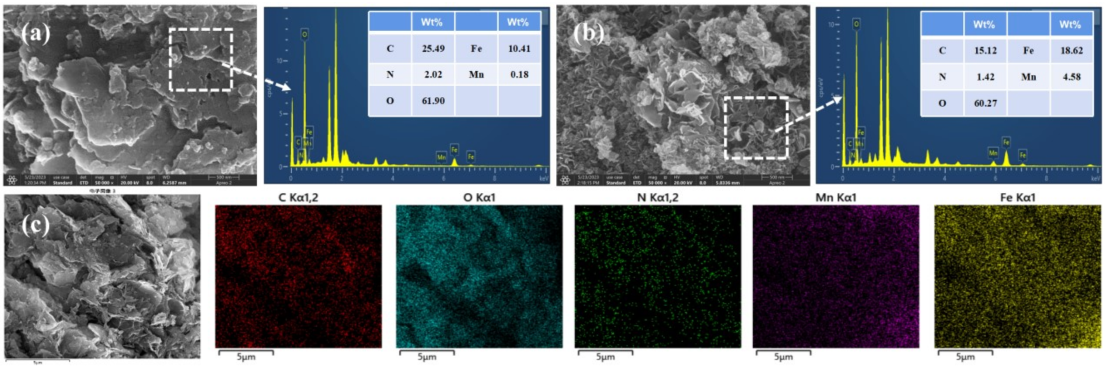


**Fig. S1** SEM-EDS analysis of BC and FMBC (a: BC, b: FMBC and c: surface elements mapping of FMBC).

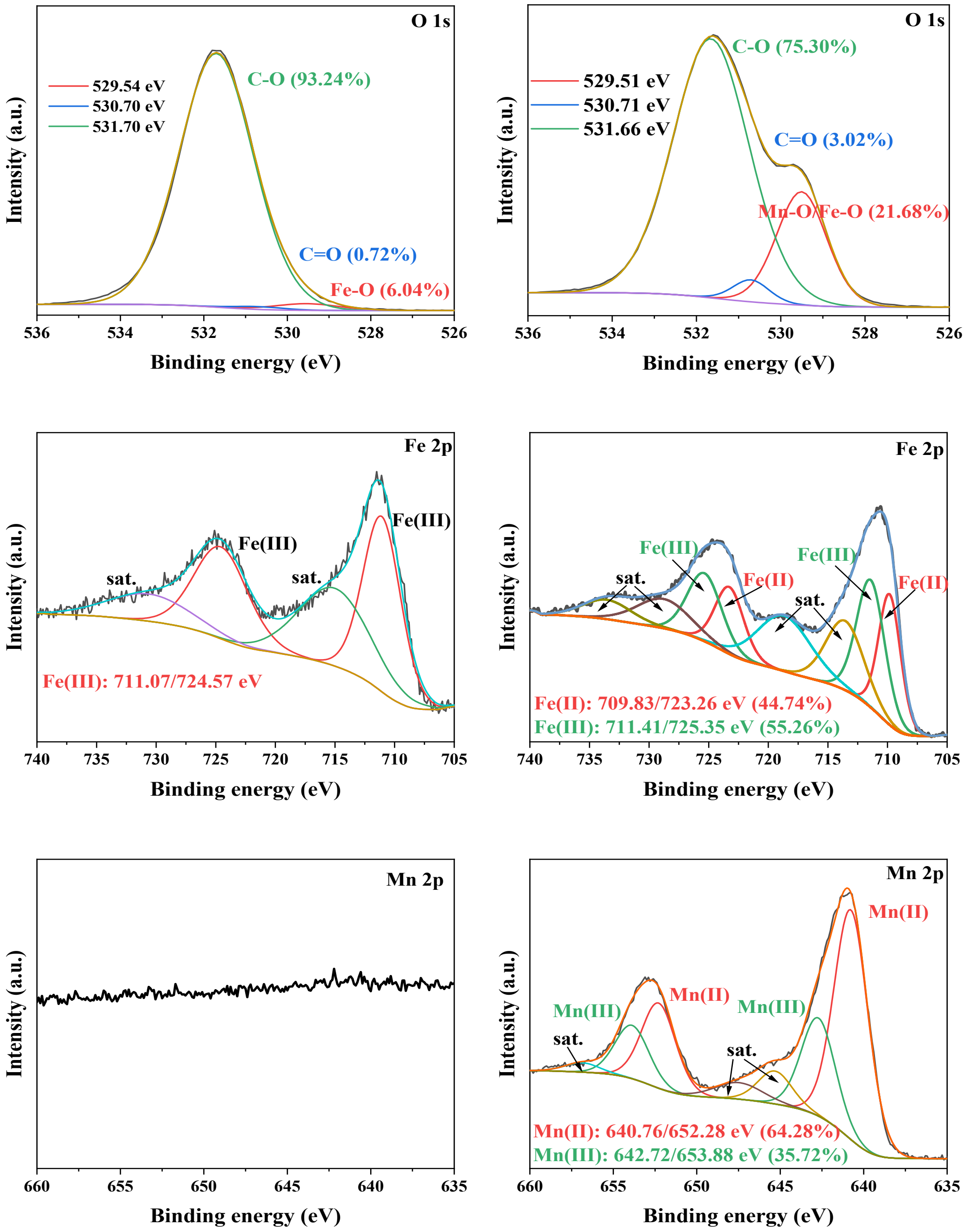

**Fig. S2** XPS High-resolution mapping of O 1s, Fe 2p and Mn 2p in BC (a, c and e) and FMBC (b, d and f).

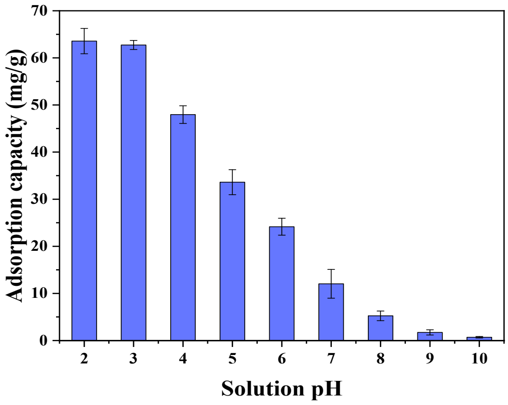


**Fig. S3** Influence of solution pH on removing Cr(VI) for BC.

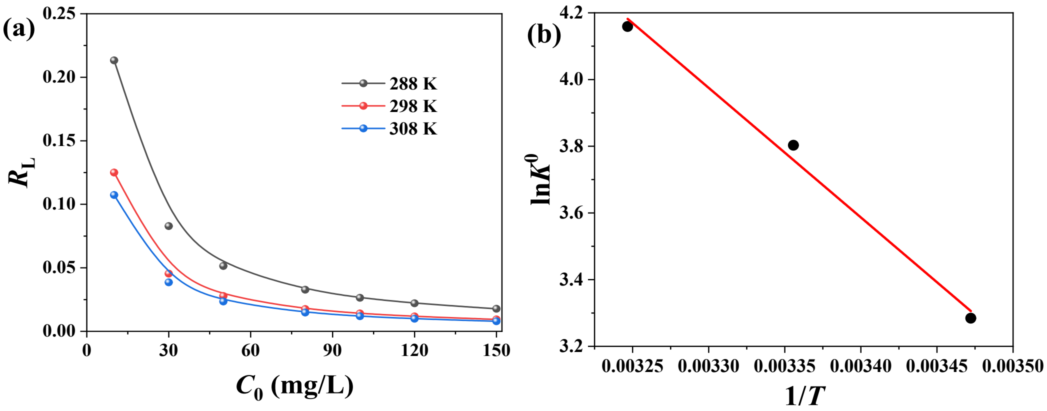


**Fig. S4** The $R_L$ value of FMBC (a) and lot of ln$K^0$ versus 1/$T$ for the estimation of thermodynamic parameters (b).

**Table S1** Properties of electroplating wastewater and mineral dissolving wastewater

| | Electroplating wastewater | Mineral dissolving wastewater |
|---|---|---|
| | Concentration (mg/L) | Concentration (mg/L) |
| pH | 2.19 | 6.76 |
| Total Cr | 43.70 | 1.54 |
| Cr(VI) | 40.21 | 1.35 |
| Cu | 2.89 | 0.32 |
| Zn | 19.48 | 0.53 |
| Pb | 3.04 | 0.89 |
| $Cl^-$ | 315.61 | 112.63 |

| | | |
|---|---|---|
| SO2- 4 | 115.94 | 68.09 |
| NH+ 4 | 60.18 | 35.42 |
| PO3- 4 | 7.03 | 2.87 |

**Table S2** Intra-particle diffusion and liquid film diffusion model for Cr(VI) removal by FMBC

| Model | Parameter | Fitting result |
|---|---|---|
| Intra-particle diffusion | $C_1$ | 12.595 |
| | $K_{d1}$ (mg/g·min$^{1/2}$) | 15.140 |
| | *R2 1* | 0.930 |
| | $C_2$ | 74.866 |
| | $K_{d2}$ (mg/g·min$^{1/2}$) | 3.475 |
| | *R2 2* | 0.929 |
| | $C_3$ | 111.626 |
| | $K_{d3}$ (mg/g·min$^{1/2}$) | 0.285 |
| | *R2 3* | 0.981 |
| Liquid film diffusion | $K_{fd}$ | -0.025 |
| | $C_{fd}$ | -0.579 |
| | $R^2$ | 0.990 |
